# Epigenetic and transcriptional control of mast cell responses

**DOI:** 10.12688/f1000research.12384.1

**Published:** 2017-11-29

**Authors:** Silvia Monticelli, Cristina Leoni

**Affiliations:** 1 Institute for Research in Biomedicine, Università della Svizzera italiana (USI), Bellinzona, Switzerland

**Keywords:** mast cell response, transcription factors, epigenetic control

## Abstract

Mast cells are tissue-resident, innate immune cells present in most tissues of the body and are important effector and immunomodulatory cells. Differentiated mast cells typically are characterized by the surface expression of the receptors KIT and FcεRI, the latter especially being important for stimulation through IgE antibodies, although these cells have the ability to respond to a wide variety of environmental signals, to which they can variably react by releasing pre-stored or *de novo*–synthesized mediators or both. Since mast cells terminate their differentiation in their tissue of residence in response to specific microenvironmental cues, each tissue may comprise unique mast cell subtypes, and responses are tailored to the danger signals that are likely to be encountered in each anatomical location. From a transcriptional point of view, these cells therefore must be endowed with epigenetic and transcriptional programs that allow them to maintain a stable identity and at the same time allow sufficient plasticity to adapt to different environmental challenges. In this commentary, we highlight some of the recent findings that advanced our understanding of the transcriptional and epigenetic programs regulating mast cell functions.

## Introduction: how many types of mast cells are there and what do they do?

**Figure 1.**
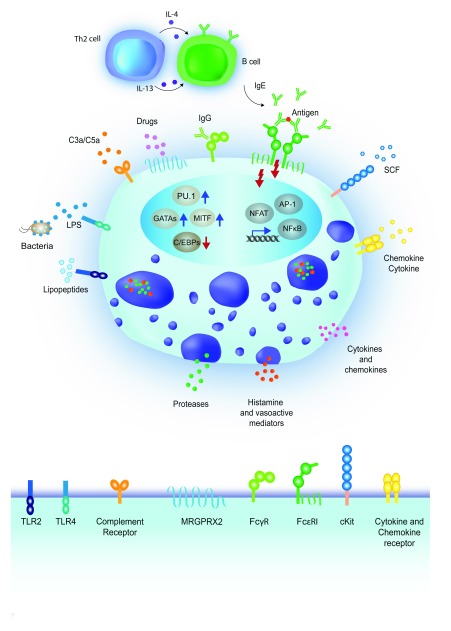
Mast cells and their receptors. Mast cells are highly reactive cells expressing a plethora of receptors with specificity toward many types of stimuli. Upon activation, mast cells can release pre-formed mediators stored in cytoplasmic granules, including proteases and vasoactive mediators, while cytokines and chemokines can be either pre-stored or *de novo*–synthesized. The most commonly studied pathway for mast cell activation includes the engagement of the high-affinity IgE receptor (FcεRI), and is dependent on the production of antigen-specific IgE antibodies by B lymphocytes, in response to interleukin-4 (IL-4) and IL-13 produced by T helper 2 (Th2) lymphocytes ^26^. Crosslinking of the FcεRI-bound IgE by antigens results in mast cell degranulation and cytokine production ^27^. Mast cells can also express surface receptors for IgG antibodies, namely FcγR, whose engagement can lead to both activating and inhibitory signals, depending on the specific receptor involved ^28^. A variety of pathogen recognition receptors (PRRs), including Toll-like receptors (TLRs), are also expressed on the mast cell surface. For example, TLR2 is activated by bacterial lipopeptides, while TLR4 is activated by lipopolysaccharide (LPS) binding. Notably, TLR-mediated activation does not usually lead to mast cell degranulation, but triggers the production of *de novo*–synthetized mediators such as cytokines and chemokines. Mast cells also express receptors for chemokines, cytokines, and growth factors, essential not only for their maturation and differentiation but also to modulate their responses. For example, the KIT receptor binds the stem cell factor (SCF), important for mast cell proliferation and maturation ^11^. The MRGPRX2 receptor is activated by a range of ligands, including inflammatory peptides and drugs associated with allergic reactions ^29^. Finally, complement receptor-mediated activation of mast cells can be induced by different complement components, such as C3a or C5a ^11^. Transcription factors such as PU.1, MITF, GATA and C/EBP family members have critical roles in regulating mast cell development and in the maintenance of cell identity ^30^, while transcription factors such as NF-κB, NFAT and AP-1 are predominantly involved in the acute regulation of inflammatory genes ^23^.

Mast cells are one of the innate immune cell types involved in the first line of defense from pathogens that attempt to breach the epithelial barriers of our organism ^1, 2^. Indeed, these cells are most notably located in vascularized tissues, including the skin, the mucosa of the lungs, and the gastrointestinal tract, where they reside primarily at the interface with the environment, namely beneath the epithelial surface. The clearest examples of immune-related responses in which mast cells play a key role are in the context of allergy ^3– 6^ as well as in immunity against parasites ^7^. However, these cells have been involved in a plethora of processes, either protective of (tissue homeostasis and wound healing) or damaging to (chronic inflammation and cancer and autoimmune diseases ^2, 8, 9^) the organism, and some of the proposed functions of these cells have also become a matter of debate ^1, 10^. The main reasons for the difficulties in obtaining concluding evidence about mast cell functions *in vivo* include the complications linked to studying tissue-resident cells as well as the complexity of mast cell phenotypes and responses. Indeed, mast cells carry a wide repertoire of receptors and can be activated by an impressive number of different stimuli ^11^ ( Figure 1), which can lead to a battery of different responses that may occur together or independently ^12^. These include degranulation with release of mediators pre-stored in cytoplasmic granules ^1, 2^, *de novo* synthesis of cytokines and chemokines, release of exosomes that may act over long distances ^13^, and even release of DNA extracellular traps ^14^. These widespread possibilities of stimuli and responses, together with the fact that these are exclusively tissue-resident cells difficult to extract in sufficient number without inducing any modification to their biology, have made the task of pinpointing their main functions remarkably challenging. For example, a study aimed at defining the human mast cell transcriptome clearly showed how mast cell transcriptional responses change dramatically upon *in vitro* culture with interleukin-4 (IL-4) and stem cell factor ^15^ as compared with freshly isolated mast cells from human skin ^16^. Transcriptional changes reflected primarily metabolic activation, most likely linked to culture-induced cell cycle progression; however, other transcriptional changes (such as the induction of genes characteristic of other lineages) were suggestive of problems in fully maintaining cell identity *in vitro*. To add to this complexity, circulating human mast cell progenitors are very rare and difficult to differentiate *in vitro*, and so far no *in vitro* system has been able to recapitulate the wide variety of phenotypes or states that are likely to exist *in vivo*. Indeed, the complexity of signals and microenvironmental cues that lead to the migration of mast cell progenitors to specific tissues remains to be fully unraveled, although these cells are clearly influenced by changes in their cytokine milieu and by the presence of activating factors ^12^.

**Figure 2.**
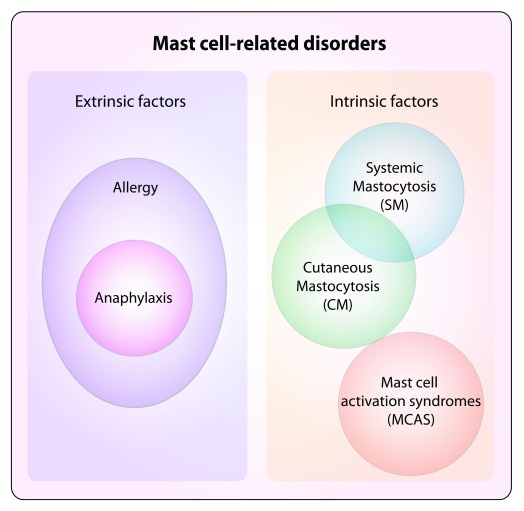
Mast cell–related disorders. Diseases associated with mast cells can broadly include disorders associated with extrinsic factors, such as the ones mediated by IgE antibodies that, acting through the high-affinity IgE receptor (FcεRI) expressed on the mast cell surface, can translate into the development of allergic reactions. Allergies are detrimental immune responses against otherwise innocuous environmental antigens, which induce the production of IgE antibodies that can activate mast cells, eventually leading to, for example, allergic rhinitis, asthma, and atopic dermatitis ^26^. Excessive allergic reactions can translate into anaphylaxis. Other disorders can instead be cell-intrinsic, due to altered biological features of mast cells that lead to uncontrolled responses (mast cell activation syndrome, or MCAS) ^31^ or excessive proliferation (systemic and cutaneous mastocytosis, or SM and CM). Potentially, all mast cell disorders can display altered activation and are broadly defined as mast cell activation disorders (MCADs), although MCAS represents a subgroup displaying mast cell activation without clonal expansion ^32, 33^.

Besides the challenges of studying mast cells, why should we care about these cells and how they are regulated? Studying mast cells has the obvious implication of a better understanding of mechanisms that are involved in allergy and asthma as well as in mast cell–proliferative diseases ^17^ ( Figure 2), and as our technological resources are improving at an unprecedented speed, we are also becoming increasingly able to gain more mechanistic details on their functions. For example, the important role of mast cells in eradicating infections by intestinal nematodes has been known for quite some time; however, the underlying mechanism was unclear. A recent study showed that mast cells respond to ATP released by intestinal epithelial cells damaged during parasite infection by secreting IL-33 ^18^. Mast cell–derived IL-33 in turn activated group 2 innate lymphoid cells (ILC2) to produce IL-13, leading to goblet cell hyperplasia and worm expulsion. Similarly, mast cells have an established role in allergic reactions, and their contribution to such responses is dependent on the acquisition of antigen-specific IgE antibodies that are bound to the high-affinity IgE receptor (FcεRI) on the cell surface. However, it was only recently elucidated that for mast cells to acquire IgE antibodies from the blood, they must display a preferential perivascular location, which enabled direct access to the blood and sampling of the intravascular lumen through cellular projections ^19^. Also, mast cells turned out to have a crucial role in orchestrating the exfoliation of epithelial cells in the bacterially infected bladder, thereby inducing an important defense mechanism aimed at reducing bacterial burden ^20^. Indeed, IL-1β produced by bacterially exposed bladder epithelial cells potently recruited mast cells to the site of infection; uptake of the released mast cell granules by epithelial cells was followed by the release of the mast cell protease Mcpt4 and caspase-1 activation, eventually leading to cell death ^20^. Although the specific signals that induced mast cell degranulation in the infected bladder remain to be elucidated, this study highlighted how mast cells can influence the outcome of many different physiological responses. Mast cells were also shown to suppress humoral and cell-mediated responses in the bladder, through the production of the anti-inflammatory cytokine IL-10, most likely in an attempt to protect the organ from excessive tissue damage ^21^. Whether the same cell can switch from a pro- to an anti-inflammatory phenotype during the course of an infection in the bladder, or instead different subsets are involved, remains to be determined. Interestingly, mast cell–derived IL-10 was also shown to modulate contact hypersensitivity reactions, although the extent of IL-10 production (and thereby the final contribution of mast cells to the amplification or attenuation of tissue pathology) appeared to be variable depending on the severity of the model of contact hypersensitivity used ^3, 5, 6^.

## Transcription factors in mast cells

Many studies have assessed the transcriptional profile of mast cells, from both human and mouse, although for the most part they used cultured cells, and only in rare cases was a comparison with *ex-vivo*–derived tissue cells attempted ^16^. Since mast cells differentiate in local tissue niches, it is probable that they adaptively develop characteristic features that allow them to best function within a given context ^8, 22^. Indeed, the fact that tissue mast cells can be quite heterogeneous (for example, from the point of view of the content of their granules) has been recognized for quite some time ^12, 22– 24^. However, from a transcriptional point of view, studying the heterogeneity of tissue mast cells implies the ability to extract them directly from various tissues and perform transcriptome analyses such as RNA sequencing. In this kind of analysis, mast cell heterogeneity may be reflected in differences in their gene expression programs and this is exactly what has been observed in murine mast cells extracted from different tissues ^25^. Indeed, mouse mast cells derived from various anatomical locations (peritoneal cavity, ear, tongue, trachea, and esophagus) displayed a high degree of heterogeneity across the different tissues, although they clustered distinctly from other profiled lymphoid and myeloid cell types, including basophils and other granulocytes. Some signature genes that specifically characterized mast cells included a number of proteases such as *Ctsg*, encoding for cathepsin G; the metalloprotease gene *Adamts9*; and *C2*, encoding for the complement component C2 of the classic C3 convertase ^25^. Among the transcription factors, *Crebl1*, *Smarca1*, and *Zfp9* appeared to be relatively specific for mast cells, although their role remains unknown, while *Mitf*, a transcription factor crucial for mast cell differentiation and functions ^34^, clearly defined mast cells from other cell types. Highlighting once again the complications associated with studies of tissue-resident cells, the authors found that incubating peritoneal mast cells in the presence of the digestion enzymes required for tissue extraction was already sufficient to alter the expression of more than 100 genes, including the gene encoding for the transcription factor Egr2. They therefore proceeded to compare enzymatically treated peritoneal mast cells with other mast cell populations enzymatically extracted from the tissues. Such analysis revealed that, overall, mast cells from the different tissues shared a core signature of 128 genes, including genes encoding for proteases or involved in metabolic pathways important for the generation of the wide repertoire of mediators that characterize mast cells. Comparative analysis of cells from the different tissues showed that mast cells from the trachea, esophagus, and tongue displayed the highest transcriptional similarity, although some specificity remained. For example, the gene encoding for the protease *Mcpt1* appeared to be relatively specific for mast cells from the esophagus. Peritoneal and skin mast cells appeared to be more divergent in their transcriptional profiles, with differential expression of a number of genes, including the adhesion molecule CD34 (which was absent in skin mast cells), the transcription factor SOX7 (increased in skin mast cells), and the integrin β2, the last of which instead was preferentially detected in peritoneal mast cells. Interestingly, peritoneal mast cells were characterized by a transcriptional signature significantly associated with mitosis, and indeed these cells appeared to undergo proliferation even in the absence of inflammation ^25^.

Apart from MITF, other transcription factors that are known to positively or negatively impact mast cell differentiation or function (or both) belong to the GATA, STAT, and C/EBP families (reviewed in 23, 30) ( Figure 1). For instance, STAT5 expression was shown to be crucial in modulating mast cell survival in response to cytokine signals ^35^, and STAT5 activity in mast cells was linked to allergen-induced dermatitis ^36^. Interestingly, several transcription factors also showed some level of crosstalk in regulating mast cell differentiation and functions: for example, C/EBPα and MITF acted antagonistically in the specification of the basophil and mast cell lineages ^37^, while STAT5 acted upstream of GATA2 in the differentiation pathways leading to either mast cells or basophils ^38^. Other transcription factors such as HES1 ^39^, EGR family members ^40, 41^, or ZEB2 ^42^ have also been associated with at least some specific aspects of mast cell biology ^23^, although their exact role *in vivo* or their detailed mechanism of action at the genomic level requires further investigation. Of note, many of the transcription factors that are involved in mast cell activation (NFAT, NF-κB, AP-1, and so on) are also more general regulators of inflammatory genes in many immune cell types, and they will not be extensively discussed here. We refer the reader to a more comprehensive review on this topic ^23^.

## Innate immune memory

The cell-intrinsic, short-term memory of an encounter with a pathogen or a danger signal ^43^ may be especially relevant for mast cells compared with very short-lived cells such as neutrophils and basophils. Mast cells are very long-lived cells, retain the ability to proliferate despite being fully differentiated ^2^, and can even replenish and modulate the composition of their granules after stimulation ^44^. The process of enhanced innate immune response against a secondary encounter with a pathogen, which was clearly defined in macrophages and other innate immune cells as “trained immunity” ^45^, could influence secondary mast cell responses after the first activation and could be important in the modulation of protective as well as allergic responses. Mechanistically, such innate immune memory is thought to be based mainly on epigenetic reprogramming, involving histone modifications, DNA methylation, and even the expression of selected microRNAs and other non-coding RNAs, which collectively contribute to the rewiring of the transcriptional program of the cell upon stimulation ^43, 45^. However, not many studies addressed the issue of cell-intrinsic, long-term changes in mast cell functional programs in response to a stimulus. For example, lipopolysaccharide (LPS) stimulation of mast cells can induce a state of unresponsiveness to a subsequent stimulation which is similar to the endotoxin tolerance described for macrophages, thereby probably representing a genuine example of mast cell short-term memory ^46^, and some crosstalk between IgE and LPS stimulation in mast cells was also reported ^47^. However, mast cells can be directly activated by many additional stimuli ( Figure 1), and whether a true trained immunity applies to mast cells for at least some of these stimuli, the relevance of such a process *in vivo*, and the underlying mechanisms are all aspects that require further investigation, especially in view of a potentially crucial role in modulating mast cell responses.

## Epigenetic control of mast cell responses

Chromatin modifications such as covalent modifications of histone tails or DNA methylation are epigenetic mechanisms of regulation of transcription, which include all of those mechanisms that influence gene expression by modulating the accessibility of regulatory regions to transcription factors without altering the DNA sequence. Epigenetic modifications are critical mechanisms that modulate the interplay of genomic sequences with environmental signals, and indeed they have a crucial role during development, in the maintenance of cell identity, in cell differentiation, and in regulating acute responses to stimuli. Because of the complex networks in which these mechanisms act, and their ability to affect the entire genome, epigenetic studies often suffer from major obstacles that hinder our mechanistic understanding of the biological role of epigenetic modifications ^48^. For instance, loss-of-function studies of DNA methyltransferase (DNMT) enzymes revealed the unmistakable importance of this epigenetic modification during development ^49, 50^; however, the molecular mechanisms underlying the embryonic or perinatal lethality observed in the absence of DNMT enzymes remain far from being firmly established. Indeed, distinguishing causality from association or direct and indirect effects of chromatin-related processes remain major challenges ^48^.

What is the role of epigenetic modifications in regulating mast cell biology? In terms of histone modifications, by assessing the role of the regulatory subunit ASXL1 of a deubiquitinase complex in hematopoietic differentiation, a recent study showed that altered levels of monoubiquitination of lysine 119 on histone H2A affected mast cell differentiation ^51^, pointing toward a key role for histone modifications in this process. Interestingly, mast cells appear to exploit an atypical epigenetic regulatory mechanism, mediated by the endogenously produced protease tryptase that not only can be secreted by the cells to affect the extracellular milieu but also can translocate into the nucleus of the producing cell, where it mediates the cleavage of histone tails, thereby modulating gene expression ^52^. While some mechanistic aspects (such as the regulation of tryptase nuclear translocation) remain to be understood, this example further highlights the potential importance of histone modifications in mast cell biology. Accordingly, histone deacetylase inhibitors appeared to impact the proliferation and viability of pathogenic mast cells derived from patients with mast cell–proliferative disorders ^53^. But in general, very few studies have investigated the role of histone modifications or histone-modifying enzymes in regulating mast cell differentiation and function. Further studies in this direction, especially in primary mast cells obtained from tissues, would certainly provide further insights about how these cells are regulated and what goes wrong during disease.

**Figure 3.**
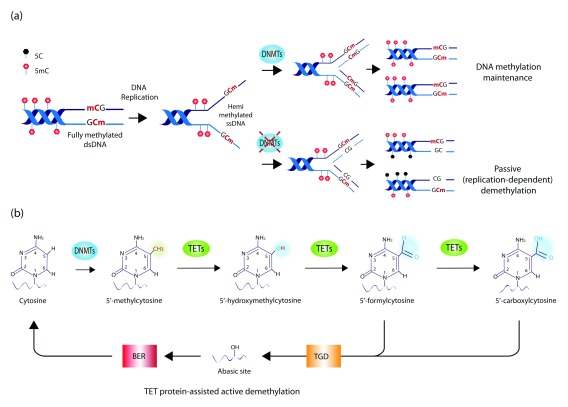
DNA methylation dynamics. ( **a**) Mechanisms of maintenance and loss of DNA methylation. During DNA replication, DNMT1 binds hemi-methylated single-stranded DNA (ssDNA) and copies DNA methylation patterns on the newly synthetized DNA strand, thereby maintaining the overall DNA methylation landscape across DNA replication and cell division. In the absence of DNMT1, such a process is impaired, resulting in the passive dilution of the methyl mark during cell division. While DNMT1 acts as the primary maintenance DNMT enzyme during cell division, DNMT3A and DNMT3B also contribute to DNA methylation as *de novo* DNMTs (namely they do not require a hemi-methylated DNA template but also can act on fully unmethylated DNA). ( **b**) DNA methylation and hydroxymethylation. DNMT enzymes methylate the 5′ carbon residue on the cytosine ring in DNA, giving rise to 5′-methylcytosine (5mC). Iterative oxidation of the methyl group mediated by ten-eleven-translocation (TET) enzymes leads to the formation of 5′-hydroxymethylcytosine, followed by 5′-formylcytosine and 5′-carboxylcytosine. The latter two modifications are recognized and excised by the thymine-DNA-glycosylase (TGD) enzyme, leaving an abasic site in the DNA, which is repaired by base excision repair (BER) mechanisms. Because this process of iterative oxidation leads to the substitution of a modified cytosine with an unmodified one, it is also termed TET protein-assisted active DNA demethylation.

As for DNA methylation, this process is mediated by DNMT enzymes that covalently link a methyl group to cytosines in the genomic DNA to give rise to 5-methylcytosine (5mC). Such modification can influence gene expression either by interfering with the binding of transcription factors and co-regulators to a given DNA sequence or by recruiting specific methyl-binding proteins ^54^. The importance of this process in transcriptional regulation is highlighted not only by the altered developmental processes observed in the absence of DNMT enzymes but also by the many examples of disease, including hematological malignancies, in which DNMT enzymes are mutated and DNA methylation is affected ^55^. Methylated cytosines in the genomic DNA can also be oxidized to 5-hydroxymethylcytosine (5hmC) by the action of the ten-eleven-translocation enzymes TET1-3 ( Figure 3). Such modification is usually enriched at enhancer elements and correlates with transcription ^56^. DNA methylation–related processes are also critical to specifically modulate mast cell differentiation and functions; indeed, we found that deletion of the mouse *Tet2* gene affected primarily mast cell differentiation and proliferation ^57^. Interestingly, while cells lacking *Tet2* were characterized by a very significant hyperproliferation compared with wild-type cells, heterozygous cells displayed an intermediate phenotype, pointing toward gene-dosage effects. These results suggest that somatic *TET2* mutations on one allele may be sufficient to predispose individuals to excessive mast cell proliferation, although in the absence of additional mutations they are insufficient to cause overt disease ^58, 59^. Unlike *Tet2* ablation, deletion of *Dnmt3a* in the mouse led primarily to unrestrained mast cell responses to stimuli, with significantly increased mast cell degranulation and activation both *in vitro* and in *in vivo* ^60^. Importantly, treatment with demethylating agents or down-modulation of the expression of another DNMT enzyme recapitulated or even exacerbated these phenotypes, at least *in vitro*, highlighting the key role of DNA methylation–related mechanisms in modulating mast cell activation ^60, 61^.

## DNA methylation in disease

Mast cell–related diseases manifest in a wide range of disorders displaying activation or proliferative dysregulation or both ( Figure 2) ^31, 62^. In particular, mastocytosis is characterized by the abnormal proliferation and accumulation of mast cells in various organs and tissues. While the exact cause of mastocytosis remains unclear, it is frequently associated with mutations in the *KIT* oncogene, most commonly an aspartic acid–to–valine substitution at codon 816, causing spontaneous activation of the KIT receptor ^62^. One of the first indications that DNA methylation–related processes might be important in mast cell biology came from the observation that decitabine, a demethylating agent used in the clinic for the treatment of myeloproliferative and myelodysplastic disorders, induced apoptosis of neoplastic mast cells, at least *in vitro* ^63^. Moreover, mutations in the gene encoding for *DNMT3A* were identified in 3 out of 26 patients with systemic mastocytosis ^64^, while *TET2* mutations were identified in at least 20% of the patients ^59, 64, 65^, and these correlated with worse overall survival ^64^. Accordingly, reduced levels of cytosine modifications were observed in patients with systemic mastocytosis ^66^, and altered DNA methylation patterns were reported in tissues and cells of patients with asthma ^67^. However, patient studies are by necessity mostly correlative, and becoming able to distinguish whether such changes are cause or consequence or simply associate with a certain phenotype can be a daunting task. For example, systemic mastocytosis patients carrying *TET2* mutations tended to be older and have higher monocyte counts ^64^; since inactivating *TET2* mutations also accumulate with age in healthy individuals ^68, 69^, the boundaries between cause and consequence remain problematic to define. Indeed, a recent mutational analysis of more than 2,500 human subjects identified mutations in *TET2* and *DNMT3A* as the most common age-associated mutations even in healthy individuals, and *TET2* mutations had a stronger impact on the steady increase in hematopoietic clonal expansion compared with *DNMT3A* mutations ^69^. While the effects of these mutations in disease and in the normal processes of aging are being uncovered, in the future further studies will certainly lead to a better understanding of the exact role of DNA-modifying enzymes and epigenetic mechanisms in regulating mast cell functions in the context of immune responses and in disease.

## Conclusions

Thanks to the development of novel mouse models and to technological advancements allowing the *in vivo* visualization of mast cells and their in-depth molecular analysis, we are learning more about the role of these cells in various models of disease and inflammatory responses, and we are also better able to dissect some of the mechanistic aspects of such responses. However, despite the great advancements in the field in recent years, many of the mechanisms and factors underlying mast cell differentiation and responses remain poorly defined. In the future, the combination of traditional *in vitro* and single-molecule studies with single-cell genomic and genome-wide approaches ^70^ will certainly improve our understanding of mast cell subsets and functions, potentially allowing us to analyze them within the physiological context of their microenvironment. Such developing tools will surely provide definite answers to many of the questions about mast cell biology in health and disease which we are just starting to address.
